# Introduced Goldfish Affect Amphibians through Inhibition of Sexual Behaviour in Risky Habitats: an Experimental Approach

**DOI:** 10.1371/journal.pone.0082736

**Published:** 2013-11-29

**Authors:** Laurane Winandy, Mathieu Denoël

**Affiliations:** Laboratory of Fish and Amphibian Ethology, Behavioural Biology Unit, Department of Biology, Ecology and Evolution, University of Liège, Liège, Belgium; Estacion Experimental de Zonas Áridas (CSIC), Spain

## Abstract

The introduction of alien species is one of the major causes of current and global biodiversity loss. The introduction of fish can be a particular threat to native amphibian populations, which are declining worldwide. One way for amphibians to persist in such altered environments is to adopt anti-predator strategies especially at the behavioural level. However, although it has been shown that avoidance behaviour may decrease the probability of being detected by a potential predator, little is known on the consequences on sexual behaviour. In this study, we tested the hypothesis that adult Alpine newts (*Ichthyosaura alpestris*) use shelters more often and exhibit less sexual activity in the presence of goldfish (*Carassius auratus*) and that they reduce sexual activity more in risky micro-habitats than in safe environments. To this end, we assessed behavioural patterns of adult newts in a replicated laboratory design. Goldfish were present in direct contact with newts in half of the tanks. Consistently throughout the study period, significantly more newts used shelter in the presence of fish than in their absence. Newts also significantly decreased their sexual activity level overall, but specially outside the shelter when they were in direct contact with fish. These results show that fish presence can affect newts in complex ways, such as through inhibition of their reproduction. Our work highlights that integrating behaviour in conservation studies is essential to understanding the patterns of coexistence and exclusion between introduced fish and amphibians.

## Introduction

Massive introductions of alien species can disrupt ecological equilibriums by creating novel contexts in which the responses of native species may be inadequate or costly [1]. This is of huge concern because these introductions into natural environments are one of the major causes of biodiversity loss around the world [2,3]. In comparison with terrestrial ecosystems, wetlands such as ponds are particularly vulnerable to alien species introduction because native species have more difficulties to escape them [4-6]. Freshwater fish are often intentionally introduced into the wild by human activities for many reasons such as aquaculture, fisheries, biological control and ornamentation [7]. These introductions were identified as one of the main threats to amphibians [3], a class of vertebrates facing a massive decline worldwide, even recently reported as the sixth mass extinction [8,9]. The detrimental impact of fish introductions on pond-breeding amphibians is understandable since they typically evolved in aquatic habitats naturally devoid of fish [10-12]. 

Perstistence of prey in an environment with predators and other organisms implies the exhibition of strategies at a behavioural, developmental, physiological and/or morphological level [13-15]. Behavioural responses occur faster than other responses as they are more plastic and reversible [16]. They are often innate and are believed to imply a limited cost on the survival and reproductive success of individuals [13]. Rapid development of behavioural responses can be efficient in reducing the impact of introduced species [17]. In this context, prey need to recognize a predator as a potential threat [18]. However, naïve prey may misidentify the threat [19] and exhibit inappropriate anti-predator responses [20,21]. For instance, the inability to recognize an introduced predatory fish as a threat has been shown in salamander larvae that consequently did not exhibit any anti-predator responses and were killed [11,22]. On the other hand, it has also been reported that salamander larvae could respond with an unnecessary anti-predator behaviour in response to the presence of a non-predatory introduced fish and died subsequent to a reduction of foraging opportunities [23]. 

Along with predatory effects, non-consumptive effects can contribute to amphibian population extirpations [24,25]. Indeed predators do not only affect prey population by direct predation [26]. Moreover non-consumptive effects can even be worse than direct predation [27]. A way to understand these effects is to depict the mechanisms involved at the behavioural level [28,29]. To avoid disturbance from potential predators or competitors, individuals can increase vigilance behaviour or shelter use and reduce activities [30]. By reducing activity [31,32] and using shelters [33-35], amphibians decrease the probability of being detected but at a possible cost of reducing foraging and reproduction, two essential fitness components [13]. The reduction of foraging activities to avoid threat is well known and reported in fishes [36], birds [37], mammals [38] and amphibians [28,39]. In contrast, little is known on the effects on breeding opportunities. Males can present conspicuous colour and displays, which attract both females and potential predators, while females put themselves in danger to protect eggs and offspring [40]. Consequently, individuals should manifest trade-offs to optimize energy allocation between vigilance to potential threat and essential activities such as reproduction [41,42]. It implies a correct risk assessment to adjust the intensity of avoidance behaviour to the level of threat [39]. By estimating this risk correctly, prey can choose to breed in safer places [40,43]. The structure of a habitat (presence of shelters) seems to be a key determinant of risk and space used in many vertebrates [44], but its importance for reproductive activities remains to be determined in amphibians. 

The aim of this study is to determine if fish presence can affect newts through an inhibition of their sexual activities. Specifically, we assessed the impact of an introduced fish, the goldfish (*Carassius auratus*) on newt behaviour. European pond-breeding newt species do not usually cohabit with native fish. We choose to test the effect of goldfish because this species is the most frequently introduced ornamental fish in the world, particularly in permanent ponds, a typical habitat for many amphibian species [7,45], including newts [46,47]. Goldfish is a predator of amphibian eggs and larvae but usually not of adults because of gape size limitations [45,48]. Despite the observation of coexistence patterns between goldfish and amphibians, previous research showed in most cases an exclusion or a significant reduction of the abundance of several newts species in ponds where goldfish were introduced [25,46,49,50]. Contrary to natural predators such as dragonflies which are often heterogeneous in time and which co-evolved with newts, well established goldfish are persistent and can therefore lead to a complete extirpation of native species [15,51,52]. We previously showed in laboratory that indirect contact with goldfish (i.e. only cues) affected adult Alpine newts (*Ichthyosauria alpestris*) by decreasing their feeding rate and increasing their use of shelter [28]. So the fact that newts exhibited an avoidance response suggests that they identified goldfish as a threat. However, it is not known in which level fish can affect sexual activities in newts. In this context, we tested the effect of goldfish on sexual activity of the Alpine newt as a function of micro-habitat use in a laboratory replicated design. We hypothesized that in presence of fish: (1) newts will avoid micro-habitats where goldfish are present, (2) that fish will inhibit courtship activity and (3) that sexual interactions will be reduced more in micro-habitats used by goldfish than in safer habitats (i.e. under shelters). 

## Materials and Methods

### Ethic statements

The aim of this study was to determine only the potential non-consumptive effects of goldfish on adult newts by observing their sexual behaviour and avoidance behaviour in shelters. Consequently, all care was taken to plan the experiment accordingly, and thus to control for the absence of wounds to newts and to allow them to hide in protected areas from fish (see the description of laboratory maintenance). This study mimics natural conditions in wild and garden ponds where goldfish are introduced and coexist with newts. It was conducted in a licensed University of Liège laboratory (LA1610429), and the research project was accepted by the university’s Animal Ethics Commission (Protocol No.1246). The collecting permit was issued by the Service Public de Wallonie (SPW), following approval by the Conseil Wallon de la Conservation de la Nature. In the laboratory, all individuals were checked and fed every day. At the end of the experiment (June 2012), all newts were released into their capture habitat following the recommendations of the capture permit. Goldfish were maintained in a large tank (250 × 70 × 80 cm) for future experiments.

### Study organism

**Figure 1:**
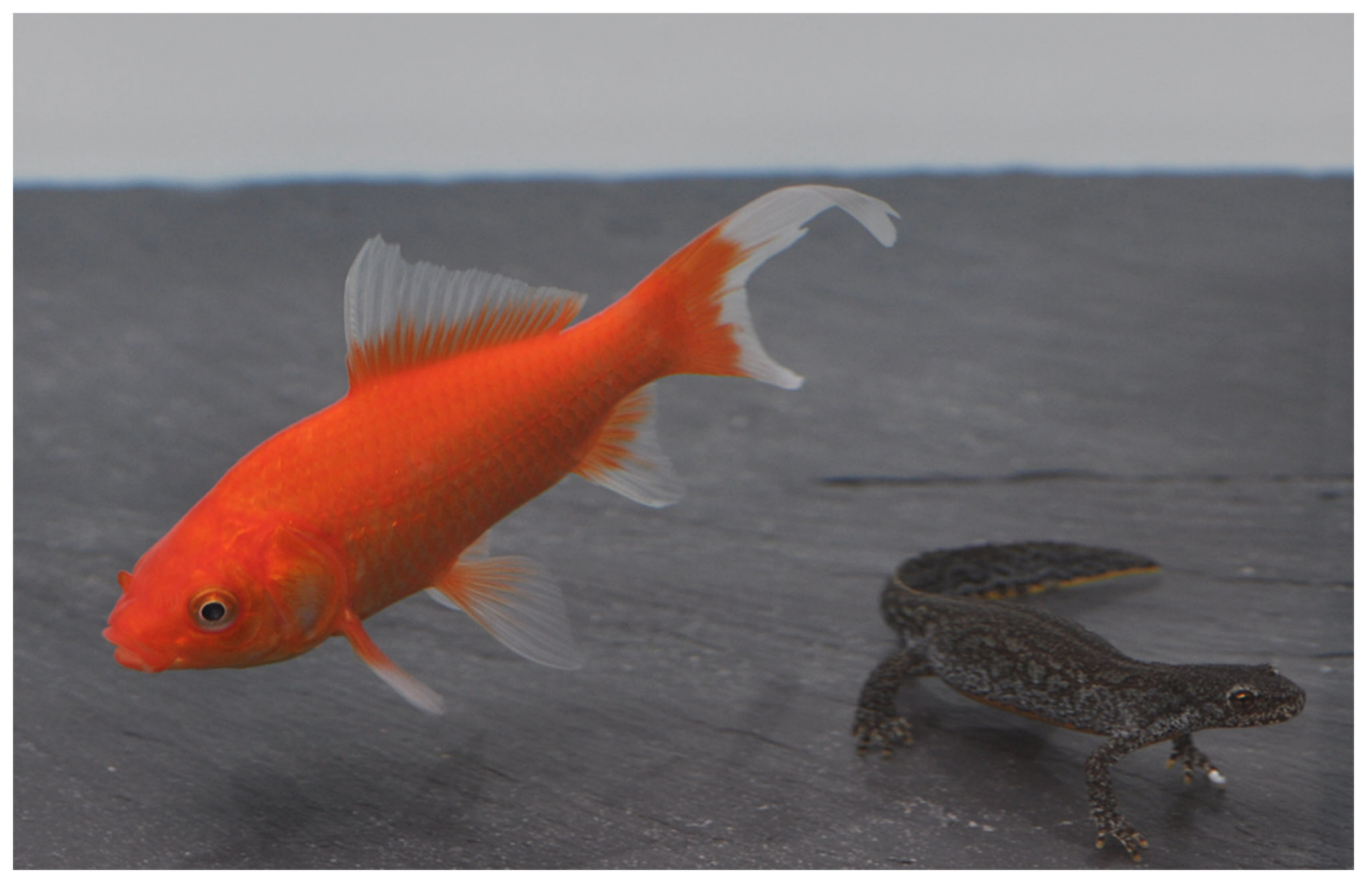
The native Alpine newt (*Ichthyosaura alpestris*) and the introduced goldfish (*Carassius auratus*).

We caught 96 (48 males and 48 females) adult Alpine newts *Ichthyosaura alpestris* (previously known as *Mesotriton alpestris* and *Triturus alpestris*; Figure 1) in Bassenge (Province of Liege, Belgium, 50°45′N–5°36′E, 70 m elevation a.s.l.) on 28^th^ and 29^th^ February and the 1^st^ March 2012. We captured newts by hand on the road during their terrestrial migration from their hibernation place to the pond where they reproduce. Because newts were caught at their first migration before they entered the pond, they had not yet reproduced that year. During the breeding season (from the end of February to the end of May in the study population), Alpine newts are aquatic. Sexual behaviour is exhibited during that time and it can last up to several months. Throughout the rest of the year, Alpine newts have a terrestrial life. Sexes are dimorphic with males smaller than females (mean ± SE total length in the studied population: 8.86 ± 0.72 cm and 10.06 ± 0.60 cm, respectively, *N* = 48 in each sex, *t*_94_ = 15.71; *P* < 0.001) and present secondary sexual traits such as a dorsal crest and a swollen cloaca in males [53]. Just after capture, we brought all newts directly to the laboratory (20-min drive) in refrigerated boxes (5–10°C; 3L) containing humid substrates (hydrophilous cotton). Goldfish were not present in the pond where the newts studied here reproduce, but Alpine newts are known to coexist with goldfish at other nearby locations [49].

### Laboratory maintenance

Males and females were kept separately in six tanks (70 × 35 cm, 30 cm water level) for 1–2 days until 96 newts were caught (1^st^ March 2012). Afterward we distributed the newts in 24 identical tanks (60 × 60 cm, 40 cm water level; 135 L) with four individuals per tank (two males and two females). All tanks were fully independent (no connection). An oxygen diffuser was placed in each tank. The bottom of the tanks was covered with pieces of slate. We provided one large shelter (20 × 60 cm) in each tank, a stone placed obliquely along one of the walls. We also placed a grid (25 mm mesh size) along the shelter to prevent fish entering the shelter (Figure 1). The part of the aquarium outside the shelter is hereafter referred to as the “open area”. The ambient air temperature was regulated to maintain water temperature at an average of 14.67°C (SE = 0.03°C). We established a photoperiod (with one Lumilux de lux 2350-lm daylight tube, L36W/12-950 and one Sylvania Professional tube, 36W DECOR183) that reflected the natural cycle of the capture location: beginning at 11 h light/13 h dark at the start of experiment and ending at 14 h light/10 h dark at the end of the experiment. Subjects were fed with 50 mg of *Chironomus* larvae per newt every day in the afternoon; after the behavioural observations to not interfere with the experiment. This food amount corresponded to the natural feeding rate of this species [54,55]. *Chironomus* larvae were placed in the shelter behind the grid so the fish could not eat them. 

**Figure 2:**
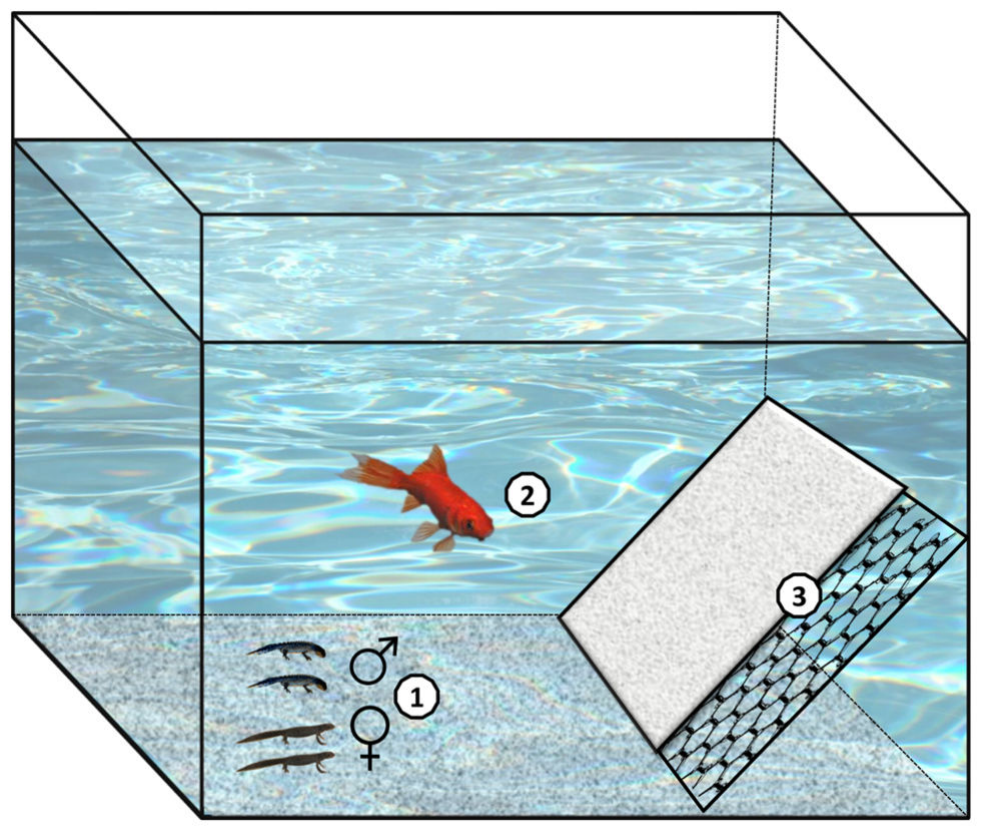
Experimental tank. 1. Four Alpine newts (two males and two females), 2. One individual goldfish (present in 12 out of 24 tanks), 3. Shelter (behind an oblique stone closed by a grid preventing fish access).

The goldfish came from a fish retailer (Blue Coral, Herstal, provider of the Aquarium of the University of Liège). We obtained them 2 months before the beginning of the experiment. They were stored in several tanks (70 × 35 cm, 30 cm water level) at an average temperature of 18°C and a photoperiod of 11h light/13h dark. At the beginning of the experiment, we placed one goldfish in 12 of the 24 experimental newt tanks as described above, so conditions applied to fish were similar to that applied to newts. The newts and goldfish were placed in tanks at the same time. Goldfish were in direct contact with newts (Figures 1 and 2) but could not go behind the grid to the shelter. Goldfish had a mean (± SE) total length of 15.38 ± 1.76 cm (*N* = 12). From the day of their arrival to the end of the experiment, they were fed with 200 mg of *Chironomus* larvae per fish every day. Goldfish received the same food as newts to avoid detection and effects of fish diet cues on newts [56]. Food was provided to the goldfish at the surface of the water so newts did not use it (Goldfish consumed *Chironomus* larvae quickly). Since both fish and newts received food at the same time and that newts received it under the shelter, there were no interaction or competition for food.

### Experimental procedure

Visual observations began the day following fish and newt transfer to the experimental tanks and were conducted by a single observer. The experimental unit was based on the 24 tanks (*N* = 12 for experimental and control tanks) (Figure 2).We used a scan sampling method [57], based on previous Alpine newt research [28,58]. Scans were replicated twenty times for each of the ten weeks and throughout 24 aquariums (i.e. a total of 4800 scans). These observations comprised five successive scans 5 min apart. So newts in each tank were scanned every 5 min during 25 min in the morning (9 a.m.) and the afternoon (2 p.m.). This procedure was replicated 2 days each week (on Mondays and Thursdays) during 10 weeks, which corresponds to the period of reproduction of the study species. From a total of 20 scans per tank and per week, we assessed the following behavioural patterns: micro-habitat use (i.e. shelter *versus* open area) and exhibition of sexual behaviour (i.e. exhibiting a courtship display). The area under shelter can be seen without difficulties through the glass of the aquariums (Figure 2). The sexual behaviour of Alpine newts occurs in water. During sexual encounters, males exhibit varied courtship displays, involving caudal movements, and can deposit spermatophores (i.e., packs of sperm) on the substratum [53,59]. To make sure that scans did not happen in pauses between successive displays (i.e. during a potential sexual behaviour that could have been overlooked), we waited up to one min when a male was close to a female to confirm the absence of on-going sexual behaviour. All courtship acts are conspicuous and cannot be overlooked [53,59]. We assessed (1) the proportion of open area use, calculated as the number of newts in each specific area (as only two micro-habitats were provided, the number of newts using the shelter was automatically deduced from this number); (2) the number of sexual activity events (calculated as the number of males exhibiting a courtship) both in the open area and under the shelter; (3) the weighted sexual activity in each micro-habitat (calculated based on the proportion of sexual activity in the open area or shelter weighted by the presence of newts in these areas). The weighted sexual activity allowed having the rate of courtship of only the newts present in specific habitat. Finally during each observation session, we also counted (4) the number of spermatophores deposited by the male in each micro-habitat (open area *versus* shelter). The presence of spermatophores is a good indication of sexual activity.

### Statistical analysis

The behavioural scores of each week were computed on the basis of 20 scans (i.e. 480 scans per week in total). The experiment lasted 10 weeks, so we had a total of 10 periods of replicates. We used a generalized linear mixed model (GLMM) assuming binomial error to test the effect of fish (fixed factor), the effect of weeks (ordinal variable, included as both fixed and random effect) and the interaction between fish and weeks on the proportion of presence in open area. We used a GLMM assuming Poisson error to test the effect of fish (fixed factor), the effect of habitat (fixed factor, shelter *versus* open area), the effect of weeks (ordinal variable, included as both fixed and random effect) and the different interactions between fish, habitat and weeks on the number of sexual activity events observed each week (count-type data). We computed the same model for the weighted variables but we used a binomial error since these data are proportions. For the number of spermatophores, we merged data over the whole study period because of low scores within each observation session. Spermatophores were counted only once because they were degraded between each observation session. We used a GLMM assuming Poisson error distribution to test the effect of fish, habitat (shelter *versus* open area) and their interaction on the total number of spermatophores. In all statistical analyses, we included aquariums as a random factor and we chose an *a priori* level of significance of 0.05. Analyses were performed in R 2.12 (www.r-project.org) using the lme4 package.

## Results

### Open area use

**Figure 3:**
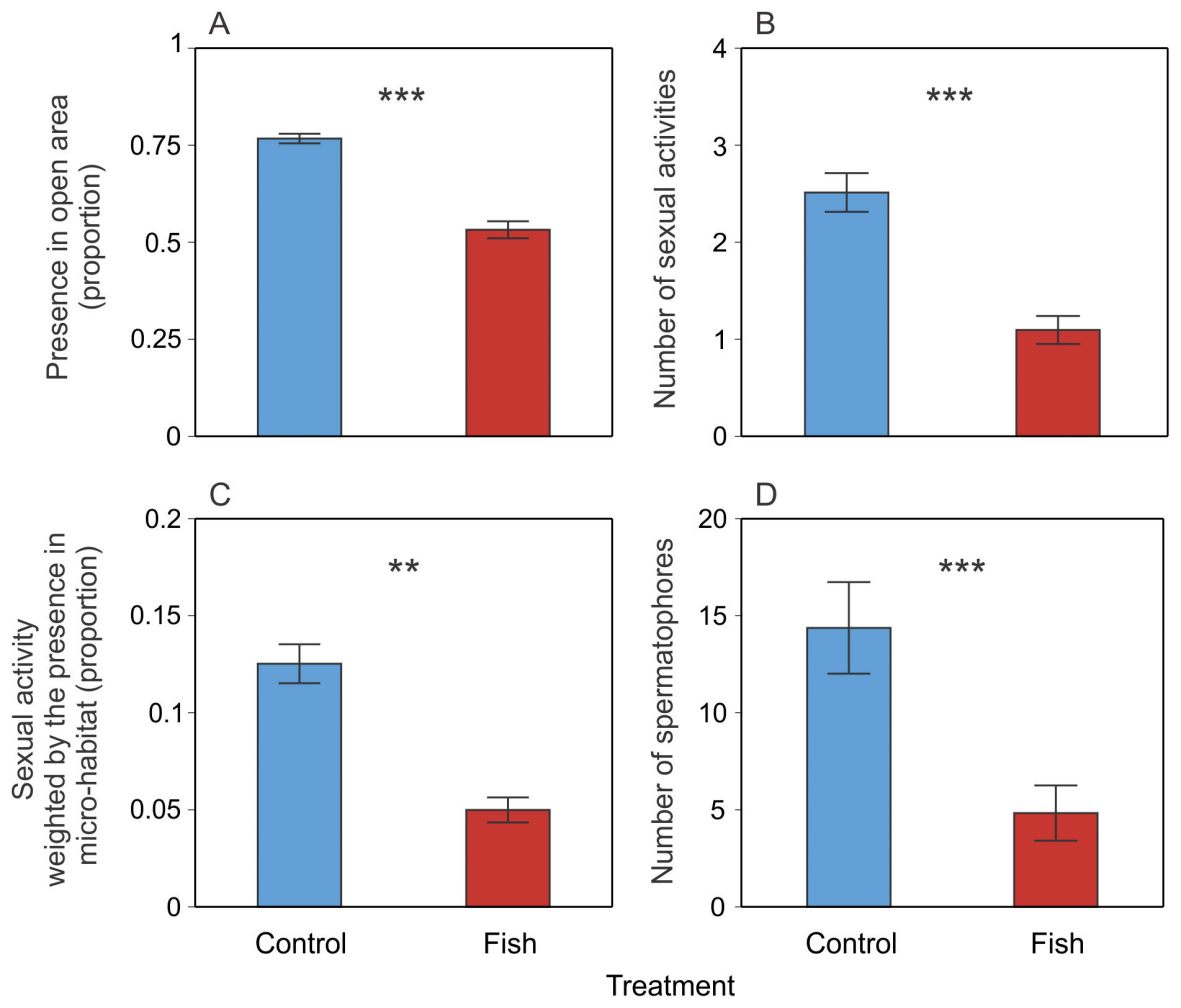
Significant effects of fish on Alpine newt behaviour (mean ± SE). The panels represent the proportion of newt presence in open area (A), the number of sexual activities (per week) (B), the sexual activity weighted by the presence in micro-habitat (proportion) (C), the number of spermatophores (per tank) (D). ** *P* < 0.01, *** *P* < 0.001 (GLMM; *n* = 12 tanks per treatment). Light blue bars: control treatment; red bars: fish treatment.

There was a highly significant effect of fish presence on the use of the open area, i.e. the micro-habitat where goldfish had access (Table 1): newts with fish used the open area less often than newts without fish (Figure 3A). There was no significant effect of weeks or of the interaction between fish and weeks on the use of the open area (Table 1).

**Table 1:** Effect of fish on Alpine newt behaviour.

Variables	Factors	d.f.	*χ^2^*	*P*
Presence in open area	Fish	1	13.276	**< 0.001**
	Week	1	3.23	0.07
	Fish × Week	1	0.09	0.76
Number of sexual activities	Fish	1	12.34	**< 0.001**
	Habitat	1	17.24	**< 0.001**
	Week	1	6	**0.01**
	Fish × Habitat	1	152.68	**< 0.001**
	Fish x Week	1	7.04	**0.008**
	Fish x Habitat x Week	1	9.15	**0.002**
Weighted sexual activity	Fish	1	8.86	**0.003**
	Habitat	1	1.35	0.25
	Week	1	3.4	0.06
	Fish × Habitat	1	1.93	0.16
	Fish x Week	1	0.19	0.67
	Fish x Habitat x Week	1	0.9	0.34
Number of spermatophores	Fish	1	8.158	**< 0.001**
	Habitat	1	2.09	0.16
	Fish × Habitat	1	120.56	**0.001**

GLMMs evaluating the effect of fish, habitat, week and their interactions on Alpine newt behaviour: presence in open area, number of sexual activity events, sexual activity weighted by the presence in micro-habitats, and number of spermatophores. Significant values are highlighted in bold.

### Sexual activity

**Figure 4:**
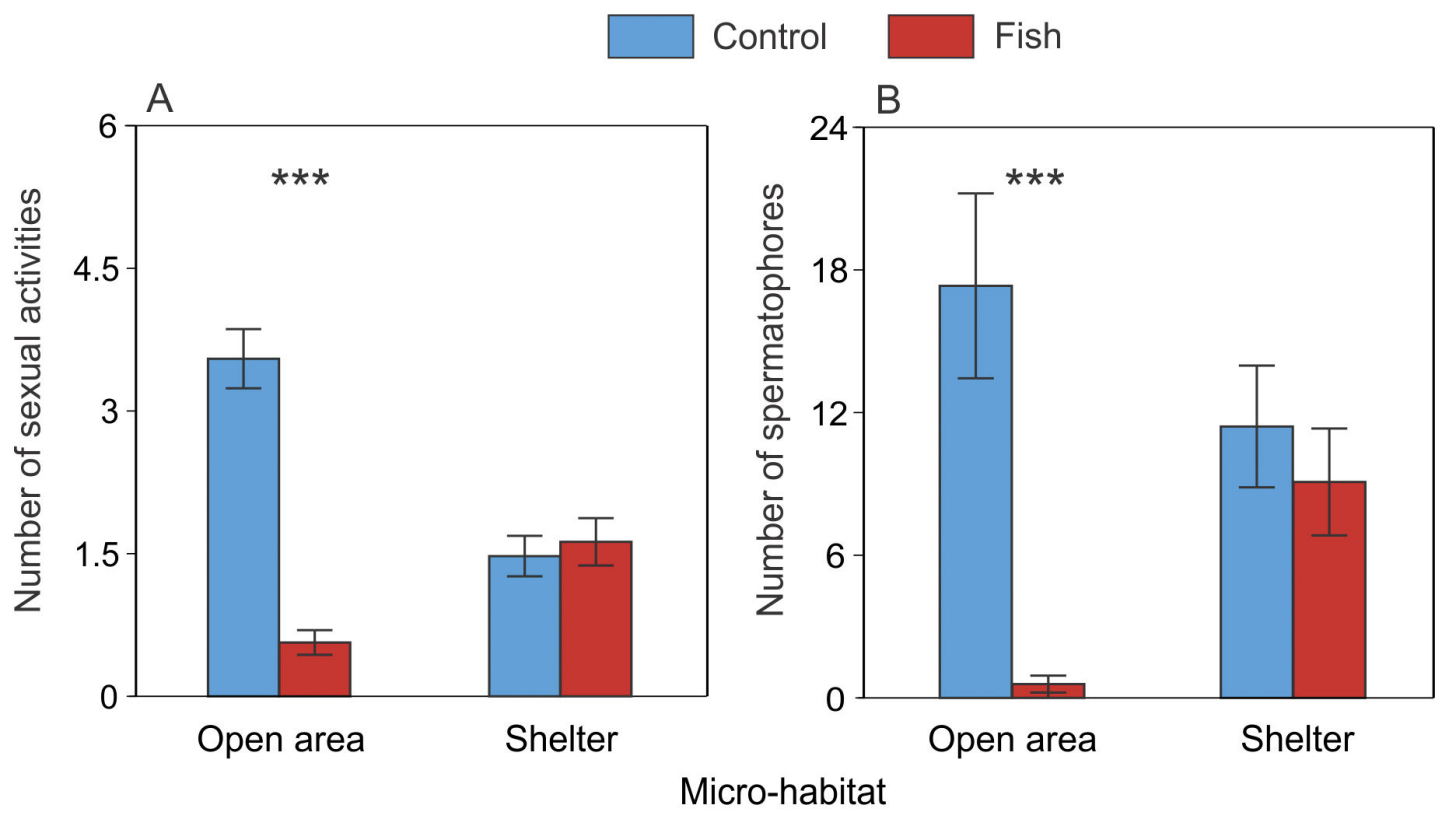
Significant interactions between the effects of fish and micro-habitat on Alpine newt behaviour (mean ± SE). The panels represent the number of sexual activities (per week) (A) and the number of spermatophores (per tank) (B). *** P < 0.001 (GLMM; *n* = 12 tanks per treatment). Light blue bars: control treatment; red bars: fish treatment.

**Figure 5:**
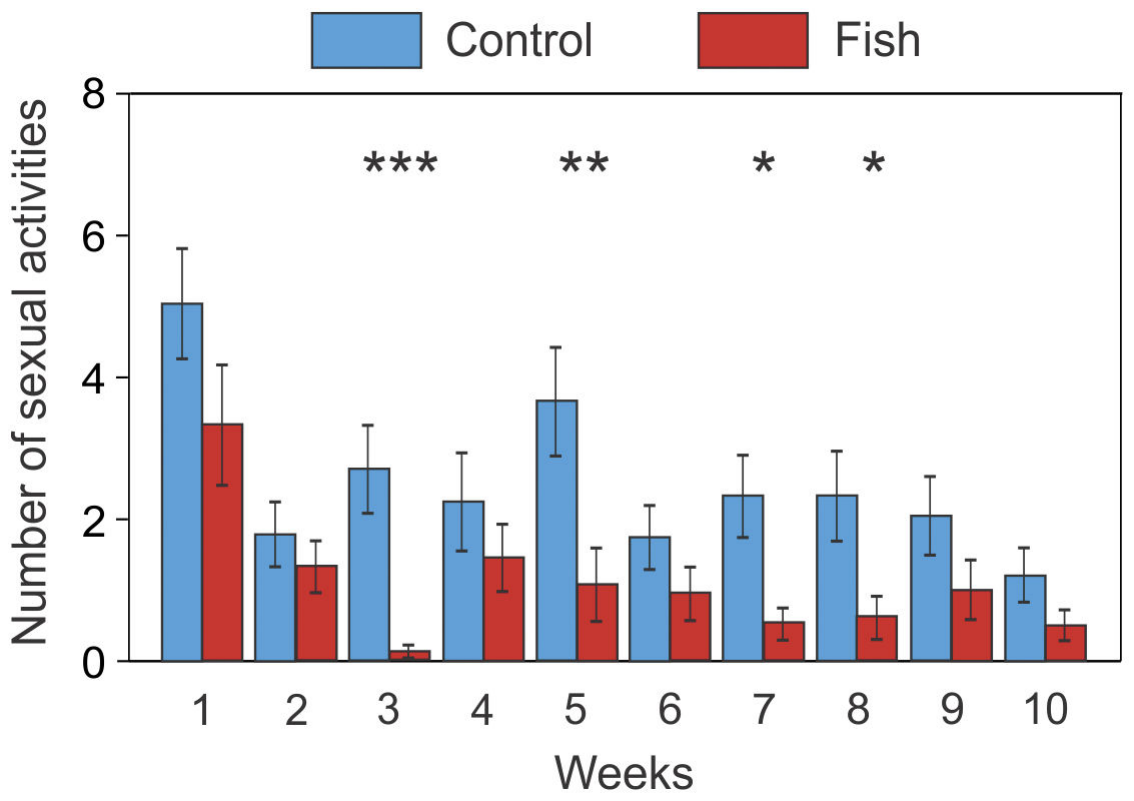
Significant interaction between the effects of fish and week on the number of sexual activities (mean ± SE). * *P* < 0.05, ** *P* < 0.01, *** *P* < 0.001 (GLMM; *n* = 12 tanks per treatment). Light blue bars: control treatment; red bars: fish treatment.

**Figure 6:**
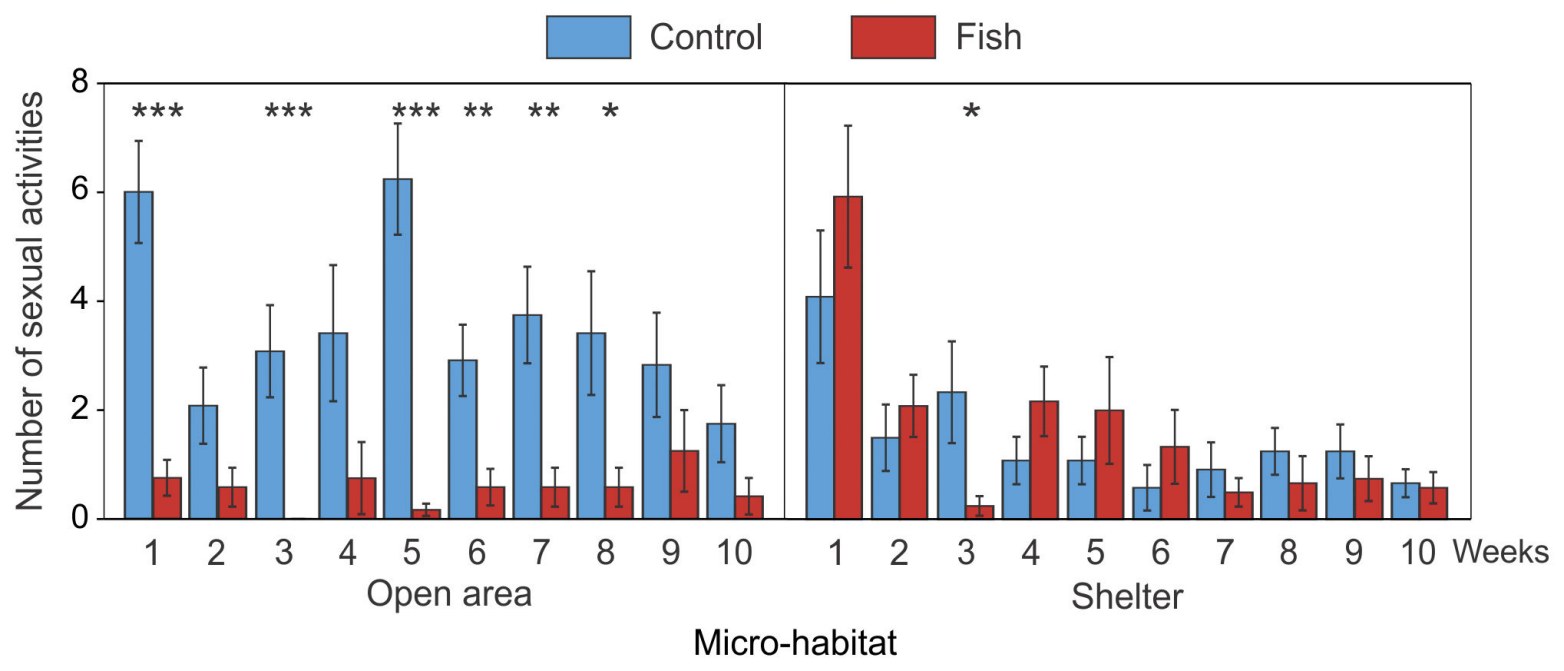
Significant interaction between the effects of fish, habitat and week on the number of sexual activities (mean ± SE). * *P* < 0.05, ** *P* < 0.01, *** *P* < 0.001 (GLMM; *n* = 12 tanks per treatment). Light blue bars: control treatment; red bars: fish treatment.

There was a highly significant effect of fish presence, habitat and weeks on the number of sexual activity events (Table 1). In aquariums with fish, we observed less sexual behaviour than in aquariums of the control treatment (Figure 3B). There was a significant interaction between fish and habitat (Table 1): we found an effect of fish on the number of sexual activity events exhibited in open area but not in shelter (Figure 4A). We also found a significant interaction between fish and weeks (Table 1, Figure 5) and a significant interaction between fish, habitat and weeks (Table 1, Figure 6).

We found a highly significant effect of fish on the sexual activities exhibited in each micro-habitat (open area *versus* shelter) when weighted by the presence of newts in these habitats: newts displayed proportionally less courtship in the presence of fish than in their absence (Table 1, Figure 3C). We did not find any significant effect of habitat and weeks as well as no significant interaction between these variables (Table 1). 

### Spermatophores

There was a highly significant effect of fish on the number of spermatophores found on the substratum of the tanks (Table 1): there were significantly fewer spermatophores in tanks containing fish than in those without fish (Figure 1D). There was no significant effect of habitat but we found a significant interaction between fish and habitat on the number of spermatophores (Table 1): there were significantly many fewer spermatophores in the open area for the fish treatment than for the control treatment but no significant difference was found in the shelter (Figure 4B).

## Discussion

In most studies, the primary reported detrimental effect of fish on native amphibians is the direct predation on adults, larvae and/or eggs [60-64]. In contrast, very few studies examined adult amphibian behaviour in response to fish presence and to our knowledge none showed an inhibition of sexual behaviour in the presence of non-predatory fish [18,65-67]. However to correctly assess and understand the consequences of interactions between species, examining such non-consumptive effects is essential [29,44]. In this study, we found that the goldfish, a species that is usually not a predator of adult amphibians such as the Alpine newt, affects them in complex behavioural ways, i.e. not only by involving a micro-habitat shift, but also by inhibiting sexual behaviour, and this more in risky than in safe micro-habitats.

In direct contact with goldfish, newts significantly avoided fish areas by using shelters more often. Habitat shift and shelter use are the most frequently described avoidance strategies when fish are introduced in natural environment [34,35,68]. However, shelters can be limited resources, which can even be depressed by fish introduction as fish can reduce vegetation in which newts hide [69], making the coexistence between fish and amphibians more difficult. Previous research showed that female red-spotted newts (*Notophthalmus viridescens*) can shift their activity and micro-habitat use in unfavorable environment and in a natural environment, this newt species avoid areas labeled with cues from predated conspecifics [70,71]. Here we show that a direct contact with fish can be enough to trigger the avoidance behaviour. This confirms that amphibians can immediately assess habitat quality through the presence of potential predators or stressors without necessarily the presence of alarm cues from predated conspecifics [72]. The proximate cues of this habitat shift remain to be determined [31,73]. In a previous experiment [28], we found a slight habitat shift in the presence of cues (all, in indirect contact between fish and newts), i.e. a much lower response than in the present configuration involving direct contacts. This suggests that newts are able to assess threats not only by the cues of fish but also by the likelihood of more direct interactions. Indeed, in direct contact, when fish were attracted by newt movement (like during sexual activities), they can touch and sometimes they try to suck the newts as if it was food. These general disturbances could explain the increase of avoidance behaviour of newts. Nevertheless we never observed any violent behaviour or chase from goldfish toward newts during the scans done in this study (2400 for aquariums involving newts and fish) or during longer qualitative observations of the fish and newts. 

 Avoidance behaviour and shelter use reduce the risk of being detected and potentially attacked by a predator [42], but it may imply a reduction in the time spent on essential activities such as reproduction [13]. Our results support this hypothesis, in showing for the first time in newts that, in the presence of fish and without predatory acts, newts exhibit sexual behaviour less often than in their absence. Such a reduction in sexual activity has already been evidenced in fish [74,75], but in newts (*N. viridescens*), it was only associated with conspecific alarm substances that signal predation [76]. Changes in reproductive behavioural patterns can be especially obvious for species that exhibit highly conspicuous coloration and courtship displays [77], as these mating traits can attract predators [74,76]. Since many potential newt predators use visual cues to predate, decreased sexual activity seems to be an appropriate anti-predatory response, but at the cost of reducing breeding opportunities and thus overall reproductive success [76].

 These results showed that newts decreased their sexual activity more in areas used by fish (i.e. in open areas) than in shelters where fish could not enter. Indeed in open area, newts, in the absence of fish, exhibited sexual activities five times more often than newts coexisting with fish. These results can be partly explained by the lower use of the open area by newts. However, considering the rate of courtship of only the newts present in the micro-habitat with fish (sexual activity weighted by the number of newts present in the associated micro-habitat), we still found that a lower proportion of newts were involved in courtship than in the situation without fish. 

Moreover, very few spermatophores were found in the open area in comparison with the situation occurring in the control groups. The presence of spermatophores is a good indication of sexual activity [77,78]. The fact that we found very few spermatophores in the open area could have two explanations. First, since in the presence of fish, newts reproduced less often in the open area, finding fewer spermatophores is predictable. Second, we also observed that goldfish foraged on spermatophores. This is not surprising since the goldfish is regarded as an occasional to exclusive detritivore [69]. This is not detrimental to newt fitness if a fish eats an “old” spermatophore not picked up by the female, but the opposite is true if this happens during the sexual encounter. Furthermore, there was no significant difference in the number of spermatophores found in the shelter where fish could not eat them. 

In response to mating risk in open areas, newts may engage in courtship in shelters inaccessible to fish if there is enough space to reproduce [79]. In the present study, the available shelter provided sufficient space for the newts to court and mate. Darkness does not affect mating success in Alpine newt, thus it cannot be accounted for differences of the number of sexual activities [77]. Our results showed no significant difference between the number of sexual activity events under the shelter in control and fish treatments. However, when sexual activity is weighted by the presence in the corresponding micro-habitat, courtship was also reduced under the shelter in fish tanks compared to control tanks. Indeed we found a main effect of fish presence on the weighted sexual activity but no interaction with habitat showing that the effect of fish was found both in open area and in shelter. Thus, newts did not make up for the reduction of courtship in the open area by mating more in the shelter.

In a natural context, the risk of predation acts on decision making during sexual interaction and prey may postpone all sexual activity until the risk is gone [79]. Indeed, animals are able to detect and respond quickly to the temporal variation of threat [80,81]. In our study, although the goldfish is assumed not to be a predator of adult newts, newts maintained an avoidance behaviour (i.e. shelter use) throughout the entire experiment (no significant interaction between fish and weeks). This is in contrast with previous experiments involving indirect contacts with goldfish where a micro-habitat shift was observed only in the first few days [28]. In direct contact, newts could experience a closer disturbance or perception of potential risk from fish throughout the experiment so they did not decrease the avoidance behaviour over time. Although some variation of sexual activities was found across time, the present results show that fish inhibited sexual activity throughout the experiment. Such time variations of sexual activities were indicative of variations in the intensity of sexual behaviour but not of the process of habituation in the presence of fish. Indeed during all the experiment, newts in contact with fish exhibited a low rate of courtship while control newts showed variation in the intensity of courtship with a decrease in the last weeks notifying the end of the reproductive period. 

The fact that a non-predatory introduced species of adult newts induced avoidance behaviour, both with respect to space use and sexual behaviour, indicates that newts perceive it as a threat. Even if we assume that goldfish are not predators of adult newts, the observed responses are not necessarily maladaptive. Indeed since goldfish can eat eggs and larvae, the impact on reproduction can be understandable [82]. Moreover the fish can disturb the sexual display by pecking newts or foraging on the spermatophores. So the reductions of sexual activities in open area and the increase of shelter use can be considered to be a suitable response to limiting risks of disturbance. Nevertheless, in the presence of fish, to balance the very low rate of courtship in open area, newts should have displayed more under the shelter, but they did not. This perhaps over-reacting of newts to non predatory fish showed that non-consumptive effects of introduced fish can strongly affect naïve amphibians in causing reproductive costs to newts. 

## Conclusions

This study showed that introduced fish can have substantial detrimental effects on amphibian sexual behaviour by strongly inhibiting their reproduction in the laboratory even if a safe habitat (i.e. a shelter) is available. Interactions between amphibians and goldfish can therefore not be only problematic at the larval stage [32,83] through consumptive effects [45], but also at the adult stage through non-consumptive effects. The reported amphibian declines after goldfish introduction in the environment requires integrating a behavioural perspective in the analyses [7,46,49,50]. Indeed, the absence of larvae may indicate both a decreased reproductive effort and a high predatory pressure on eggs or larvae [66]. Moreover amphibians can choose the breeding site as a function of predation risk [84] and can also perceive the presence of predators that eat their offspring [85]. Studying the impact of introduced species on the behaviour of a native population can contribute to a better understanding of their complex interactions and can bring some explanation of coexistence or exclusion patterns observed in the field [28]. Our results of increased use of shelters in the presence of fish and the lower inhibition of sexual activity inside than outside, indicates that shelters can be essential components to allow subsistence of newts in cohabitation with fish. It will be very useful to transfer this kind of observations into a field or mesocosm setting with other variables of natural environment. Indeed the study of behavioural mechanisms may help identify proximate causation and develop predictive models, which can allow us to understand how animal populations will respond to anthropogenic change [86]. Consequently, recognizing animal behaviour as a valid component of conservation biology can provide solutions to specific conservation management concerns [87].
